# Combination of environmental stress and localization of l-asparaginase in *Arthrospira platensis* for production improvement

**DOI:** 10.1007/s13205-014-0215-z

**Published:** 2014-04-13

**Authors:** Asep A. Prihanto, Mamoru Wakayama

**Affiliations:** Department of Biotechnology, Graduate School of Life Sciences, Ritsumeikan University, 1-1-1 Nojihigashi, Kusatsu, Shiga 525-8577 Japan

**Keywords:** *Arthrospira platensis*, Asparaginase, Stress, Production, Localization

## Abstract

The diverse applications of l-asparaginase have led us to explore new sources of this enzyme. *Arthrospira platensis* has been scarcely reported as a new candidate for l-asparaginase production. In the present study, we localized l-asparaginase in *A. platensis* and enhanced its production. Enzyme localization was conducted by culturing cells in SOT medium and extracting the enzymes from different parts of the cell. The Taguchi method (factors studied: nitrogen, iron, sodium chloride, and temperature shock) using an L9 orthogonal array was designed for improving l-asparaginase production. The highest specific activity of l-asparaginase was found in subcellular, cytoplasmic extracts (0.166 ± 0.029 U/mg). Optimization data revealed that the highest production of l-asparaginase (0.275 ± 0.005 U) was attained by NaNO_3_, NaCl, and FeSO_4_·7H_2_O at concentrations, 1.875 g/l, 0.25 M, and 0.0075 g/l, respectively, with 1-h temperature shock at 22 °C in the dark. Results revealed more than twofold higher production of l-asparaginase than that under the normal condition. In summary, l-asparaginase appeared dominantly in the cytoplasmic region and its production could be induced by employing combined stress conditions with a Taguchi experimental design. To our best knowledge, this is the first report on l-asparaginase production in cyanobacteria of the subclass Oscillatoriophycideae.

**Electronic supplementary material:**

The online version of this article (doi:10.1007/s13205-014-0215-z) contains supplementary material, which is available to authorized users.

## Introduction

l-Asparaginase (l-asparagine amidohydrolase, EC 3.5.1.1) is an important enzyme that has applications in two main sectors: in medicine, the enzyme is used routinely for the treatment of acute lymphoblastic leukemia (Verma et al. 2007). Inhibition of leukemic cells results from depletion of the circulating pools of asparagine in the cell due to the hydrolysis of l-asparagine by l-asparaginase (ASNase). In food technology, ASNase is a potent mitigating agent for reducing the acrylamide contained in processed food. Acrylamide is suspected of being a carcinogen (Ciesarová et al. 2006; Hogervorst et al. 2008; Bongers et al. 2012).

ASNase is widely distributed among microorganisms. ASNase-derived microorganisms can cause anaphylactic shock and trigger the production of anti-ASNase antibodies when administered for a long term, yet they are still used in treatments. *Erwinia carotovora*, *E. chrysanthemi*, and *Escherichia coli* are well-known sources of ASNase (Mashburn and Wriston 1964; Pieters et al. 2011). Until now, ASNase derived from *Aspergillus niger* and *A. oryzae* is a well-known source for mitigating the effects of acrylamide because of its effectiveness and safety (Blumenthal 2004; Hendriksen et al. 2009).

Because of its potential in medicinal and food, research regarding microalgae has been conducted for a long time, and *Arthrospira platensis* may be the most studied microalgae. *A. platensis* is not only rich in nutrients but also contains several beneficial metabolites (Belay 2002). Currently, there is no specific report regarding production of ASNase in this blue-green microalga except for its proteomic data under temperature stress (Hongsthong et al. 2008; Kurdrid et al. 2011). Hence, the characterization and physiological roles of this enzyme in *A. platensis* had not been considered.

Optimization of ASNase production is important before industrial-scale production can be considered. Cells respond to environmental changes to ensure their survival. The medium and environmental conditions of the culture lead to modifications in cellular metabolism (Morgan-Kiss et al. 2006; Mosier et al. 2013). We hypothesized that ASNase can be expressed optimally in *A. platensis* under appropriate conditions. In addition, ascertaining the usability of ASNase from *A. platensis* in medical and food-technology applications is important.

Knowledge regarding the locations of ASNase is important to design an accurate extraction method as well as to confirm the role of this enzyme in *A. platensis*. In the present study, we investigated ASNase activity in all cell compartments. Furthermore, we investigated the appropriate culture conditions for optimizing ASNase production.

## Materials and methods

### Reagents

Nessler reagent was purchased from Nacalai Tesque (Kyoto, Japan). Trichloroacetic acid and Folin–Ciocalteu’s phenol reagent were from Wako Pure Chemicals (Tokyo, Japan). The other reagents were chemically pure grades of commercial products.

### Strain and culture conditions

*A. platensis* NIES-39 was grown in standard SOT medium with slight modification (Ogawa and Terui 1970). The composition of the medium was as follows: 16.8 g/l NaHCO_3_, 0.5 g/l K_2_HPO_4_, 2.5 g/l NaNO_3_, 1 g/l K_2_SO_4_, 1 g/l NaCl, 0.2 g/l MgSO_4_·7H_2_O, 0.04 g/l CaCl_2_·2H_2_O, 0.01 g/l FeSO_4_·7H_2_O, 0.08 g/l Na_2_EDTA, 0.03 mg/l H_3_BO_3_, 0.025 mg/l MnSO_4_·7H_2_O, 0.002 mg/l ZnSO_4_·7H_2_O, 0.0079 mg/l CuSO_4_·5H_2_O, 0.0021 mg/l Na_2_MoO_4_·2H_2_O in distilled water with additional 1 ml micronutrients (2.86 g/l H_3_BO_3_, 2.5 g/l MnSO_4_·7H_2_O, 0.22 g/l ZnSO_4_·7H_2_O, 0.079 g/l Na_2_Mo_4_·2H_2_O). Cells were cultured in a 500 ml Erlenmeyer flask containing 200 ml medium with reciprocal shaking at 150 rpm and 30 °C. The flask was illuminated by fluorescent white lamps providing a total intensity of approximately 130 µmol photons m^−2^ s^−1^. Cells were harvested in the mid-logarithmic phase of growth (OD_730_ = 1.00).

### Enzyme extraction

Cells were passed through filter paper for separation of cells from the medium. The medium was used as the ASNase-extracellular fraction (A). To extract the enzyme in different locations of the cell, the method described by Necinova et al. 1974 was modified and used. Filtrated cells were washed with 10 mM potassium phosphate buffer (KPB) at pH 7, resuspended in 8 ml buffer and mixed directly with the solution for periplasmic extraction (4 ml of 40 % sucrose, 8 ml of 10 mM KPB at pH 7 and 2 ml of 0.8 mg/ml of lysozyme). The suspension was incubated for 25 min at 30 °C with gentle agitation. Further, 8 ml of 10 mM KPB (pH 7) and 2 ml of EDTA (18 mg/ml) were added to the suspension. The suspension was incubated further at 30 °C with gentle agitation for 40 min. To obtain a periplasmic sample (B), the solution was centrifuged at 10,000 rpm for 20 min to procure the supernatant. To the pellet, which contained spheroplasts and remaining cells, 12 ml of ice-cold water was added. Before centrifugation of the solution for 20 min at 10,000 rpm at 4 °C, the suspension was incubated for 10 min in ice-cold water with reciprocal shaking. Supernatant I was collected and the centrifugation procedure was repeated for the pellet to procure supernatant II. Supernatants I and II were combined and centrifuged for 30 min at 12,000 rpm at 4 °C. The obtained supernatant was the cytoplasmic sample (C). To the pellet, which contained the cell membrane, 10 ml of 10 mM KPB (pH 7) was added. The suspension was centrifuged at 12,000 rpm for 30 min at 4 °C. A membrane-bound preparation (D) was obtained by the addition of 4 ml of 10 mM KPB (pH 7) to the pellet.

### Taguchi experimental design

The stress conditions affecting the enzyme yield were optimized by the Taguchi method using an L9 orthogonal array. The factors studied in this study consist of nitrogen (N), iron (Fe), and sodium chloride (NaCl) along with temperature stress at 22 °C in dark for varied duration (1, 2, and 3 h). This temperature was applied to give enough stress, but still allowed *A. platensis* to grow. N and Fe stresses were conducted by reducing the content of NaNO_3_ and FeSO_4_·7H_2_O from the original amount. NaCl stress was implemented by increasing the concentration of NaCl in standard SOT medium. Dark temperature stress was initiated after growth reached an optical density at 730 nm (OD_730_) of 0.8–1.2 at 30 °C. The layout of the L9 orthogonal array as well as levels of the factors studied is presented in Tables 1 and 2. All calculations and analyses were performed using Qualitek-4 software (NUTEK Inc. USA). The effect of these factors was analyzed on the basis of the signal-to-noise ratio (S/N).

**Table 1:** Condition of stress factors and its level in Taguchi method design

No.	Factor	Level 1	Level 2	Level 3
1	Nitrogen (%)	25	50	75
2	NaCl (M)	0.25	0.50	0.75
3	Fe (%)	25	50	75
4	*T* duration (h)	1	2	3

Duration of dark temperature stress (*T* duration) was set up on the basis of the incubation time after temperature shift by referring to the report (Deshnium et al. 2000)

**Table 2:** Taguchi’s experimental design using L9 (3 × 4) orthogonal array

Exp	Factor no.				Production (U)*
	1	2	3	4	
1	1	1	1	1	0.091 ± 0. 004
2	1	2	2	2	0.011 ± 0.027
3	1	3	3	3	0.018 ± 0.007
4	2	1	2	3	0.117 ± 0.037
5	2	2	3	1	0.063 ± 0.020
6	2	3	1	2	0.072 ± 0.039
7	3	1	3	2	0.177 ± 0.007
8	3	2	1	3	0.081 ± 0.008
9	3	3	2	1	0.130 ± 0.041
Validation	3	1	3	1	0.275 ± 0.005

The number (1, 2 and 3) below each factor no. (1–4) indicates the level of each factor described in Table 1

* The values represent the mean ± SE (*n* = 3)

### Protein and ASNase assay

Proteins were assayed using the Lowry method using egg albumin as a standard (Lowry et al. 1951). ASNase activity was measured by the Nessler method (Imada et al. 1973). A mixture of 150 μl of crude enzyme, 50 μl of 1 M KPB (pH 7), 200 μl of deionized-water, and 100 μl of 150 mM l-asparagine was incubated for 30 min at 30 °C. To the reaction mixture, was added 125 μl of 20 % trichloroacetic acid (TCA) to terminate the reaction. The mixture was then centrifuged at 2,000 rpm for 15 min. To the 450 μl of pipetted supernatant, 125 μl of the Nessler solution was added. The solution was maintained for 15 min to allow the appropriate reaction between the Nessler solution and ammonia. The results were obtained at OD_480_ nm. One unit of enzymatic activity was defined as 1 μmol of ammonia per minute under described condition.

## Results

### Distribution of ASNase

**Fig. 1:**
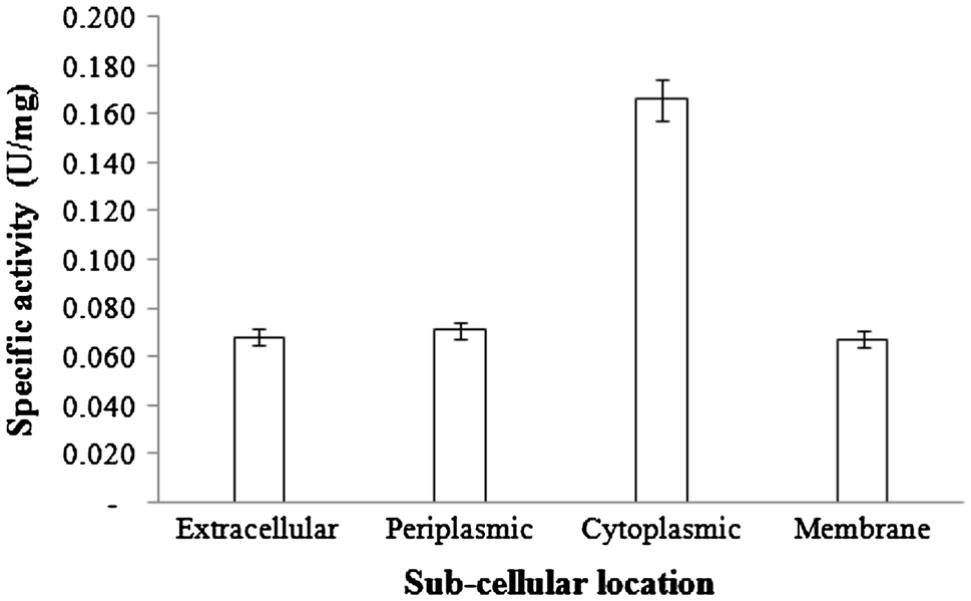
Subcellular l-asparaginase activity of *A. platensis* NIES-39. The values represent the mean ± SE (*n* = 3)

We examined the distribution of ASNase in *A. platensis* cells by measuring its enzymatic activity. Enzyme activity was detected in all parts of the cell. The calculated enzyme-specific activity was 0.067 ± 0.004–0.166 ± 0.029 U/mg. Extracellular, periplasmic fluid and membrane-bound extracts did not exhibited high ASNase-specific activity (Fig. 1). In contrast, the cytoplasmic extract exhibited high specific activity (0.166 ± 0.029 U/mg). Extracellular samples contained high protein content, and they exhibited similar specific activity to that of periplasmic and membrane-bound extracts.

### Individual factors affecting ASNase production

**Fig. 2:**
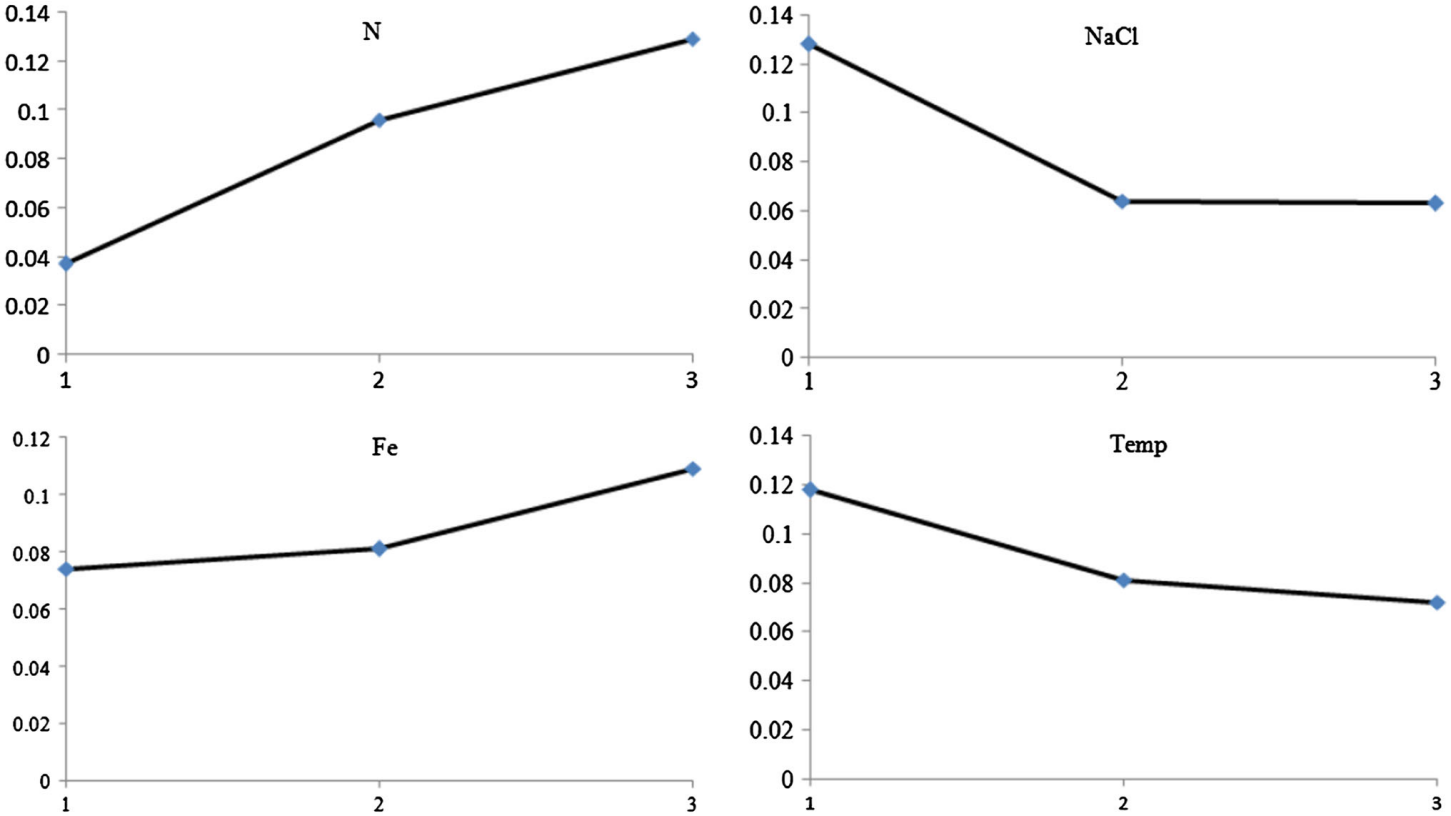
Impact of each factor to *A. platensis* ASNase production. The *horizontal axis* represents the experimental factor level. The *vertical axis* represents the ASNase production expressed in total activity (unit)

Optimization of production by combined stress was expressed as total enzyme activity. The preliminary culture condition using standard SOT medium at 30 °C did not produce a high yield of ASNase, with a yield of only 0.127 U noted in the cytoplasmic region. Several modifications with regard to the content of SOT medium and culture conditions were employed to trigger high production of ASNase in *A. platensis*. Our data revealed that these cells responded to the combination of factors in different ways (Table 2), whereas the individual factors examined suggested different effects on ASNase production (Table 3; Fig. 2).

**Table 3:** Analysis of variance (ANOVA)

#	Interaction factor	DOF (*f*)	Sum of square (S)	Variance (V)	*F*-ratio (F)	Pure sum (S′)	Percent P (%)
1	Nitrogen	2	0.025	0.012	252.893	0.025	55.405
2	NaCl	2	0.012	0.110	128.904	0.012	28.133
3	Iron	2	0.001	0	17.624	0.001	3.658
4	Duration temp	2	0.005	0.002	50.711	0.004	10.934
	Error	9	−0.001	−0.001			1.872
	Total	17	0.045				100.00

The impact, variation, and contribution of each individual selected factor were assessed by analysis of variance (ANOVA). The calculated *F*-ratio suggested that all parameters were significant at 95 and 99 % confidence intervals. Among all selected factors, N maximally affected ASNase production (55.40 %), followed by NaCl (28.13 %). Fe had a minor contribution in ASNase production (3.65 %). In this study, 83.53 % contribution was achieved with only N and NaCl stress factors. These data suggested that N and NaCl played an important role in ASNase production in *A. platensis*.

N noticeably affected the enzyme production. High amount of N in the medium generated the higher ASNase yield (Fig. 2). The highest production of ASNase (0.129 U) was obtained using 75 % N. In contrast, Fe modification had little effect upon ASNase production. A low amount of Fe in the medium failed to elicit a noteworthy impact. SOT medium containing only 25–75 % Fe yielded almost similar results with regard to ASNase production. All modification of Fe contents generated ASNase in the range 0.085–0.011 U (Fig. 2).

High NaCl content in the medium positively affected the yield of ASNase. *A. platensis* cells exhibited exhilarating responses when experiencing high sodium stress compared with the 0.017 M NaCl found in standard SOT medium. SOT medium containing 0.25 M NaCl was optimal with regard to the amount of NaCl in the medium for eliciting high production of ASNase (0.132 U). However, an excess of NaCl resulted in decreased production of ASNase. ASNase productions in *A. platensis* were the same at 0.5 M of NaCl and 0.75 M of NaCl (highest concentration).

The duration of temperature stress followed the same trend as that observed for NaCl experimental data. Incubating cultures at 22 °C for 1 h elicited the best results for ASNase production (0.117 U). In contrast, subjecting *A. platensis* cells for 2 h in temperature stress conditions decreased ASNase production, with the lowest production (0.065 U) observed for 3-h temperature stress. Longer durations of temperature stress resulted in further decrease in ASNase production. Consequently, the influence of each factor, including N, NaCl, duration of temperature stress, and Fe stress, on ASNase production was found to be 55.4, 28.1, 10.9, and 3.6 %, respectively (Table 3).

### Interaction and optimization of factors in ASNase production

The Taguchi method using the L9 orthogonal array, enabled the analysis of the influence of each factor and its interaction. In addition, the optimum condition for producing ASNase could be readily ascertained using the Qualitek-4 software. In the Taguchi method, the interaction of individual factors was estimated using the perceived severity index (SI) value. The analyses assisted to provide better understanding of the overall ASNase production process in *A. platensis*. The interaction of NaCl and duration of temperature stress represented the highest SI value of 58.13 %. In fact, Fe depletion exhibited the least significant effect upon ASNase production (3.6 %), when combined with N stress; thus, contributing to the second highest SI (37.49 %, Table 4). These data revealed that interactions of parameters could have different effects on ASNase production compared with individual parameters.

**Table 4:** Severity Index of DOE

#	Interaction factor pairs (order based on SI)	Columns	SI (%)	Col	Opt
1	NaCl × duration temp	2 × 4	58.13	6	[1,2]
2	Nitrogen × iron	1 × 3	37.49	2	[3,3]
3	Iron × duration temp	3 × 4	30.27	7	[3,2]
4	NaCl × iron	2 × 3	29.06	1	[1,3]
5	Nitrogen × NaCl	1 × 2	23.79	3	[3,1]
6	Nitrogen × duration temp	1 × 4	10.09	5	[3,2]

The predicted optimum condition for high production of ASNase in *A. platensis* was examined using modified SOT medium containing 75 % N (1.875 g/l NaNO_3_), 0.25 M NaCl, 75 % Fe (0.0075 g/l FeSO_4_·7H_2_O), and 1-h temperature stress in dark. The total contribution from all factors was 0.11 U. In this condition, the expected production of ASNase was 0.20 U (Table 5).

**Table 5:** Optimal condition and their performance in ASNase production

Factor	Level description	Level	Contribution*
Nitrogen (%)	75	3	0.039
NaCl (M)	0.25	1	0.037
Fe (%)	75	3	0.013
*T* duration (h)	1	1	0.022
Total contribution from all factor			0.11
Current grand average performance			0.09
Expected result at optimum conditions			0.20

* The values are expressed as total activity (unit)

### Validation of selected factors

Experimental validation of our prediction using SOT stress medium containing 75 % N, 0.25 M NaCl, 75 % Fe with 1-h duration in dark and temperature stresses was conducted. The maximum amount of ASNase production (0.275 ± 0.005 U) was achieved under this condition (Table 2). The obtained value was higher than the predicted production (0.20 U, Table 5). Furthermore, ASNase production was approximately 2.17-fold greater than that compared with production by culturing *A. platensis* in standard SOT medium at 30 °C in the absence of temperature stress.

## Discussion

To examine the location of ASNase in *A. platensis* cells, ASNase activities in extracellular and subcellular fractions were measured. ASNase activities were observed in several locations, with the highest specific activity being exhibited in cytoplasmic extracts (0.166 ± 0.029 U/mg). *A. platensis* NIES-39 contains two genes encoding putative ASNase, designated with entry names of NIES39_A07830 and NIES39_E04380 in the KEGG (Kyoto Encyclopedia of Genes and Genomes) data base (http://www.genome.jp). On the basis of the nucleotides sequence data, we analyzed the subcellular location of ASNase using an online server for prediction of enzyme location (Yu et al. 2006, 2010; Magnus et al. 2012). The result of analysis was presented in supplementary material 1. The CELLO subcellular localization predictor system predicted that both ASNases were in the cytoplasmic area. Furthermore, MetaLocGramN analyses suggested that both ASNases were expressed in the cytoplasmic area with 65–85 % degrees of confidence. pSORTb predicted that both enzymes were in the cytoplasmic area and that existence of these enzymes in other regions was also possible, but only in minor amounts.

The relatively large amount of protein in the extracellular fraction was probably because of the liberation of intracellular proteins. Moreover, liberation of cytoplasmic proteins probably occurred in dead cells during culture and excessively harsh agitation during *A. platensis* culture. Agitation during culture can affect the integrity of cell membranes, particularly non-covalent protein interactions in cell membranes; therefore, they become “leaky”, and can result in release of cytoplasmic proteins, including ASNase (Mader 2000).

Stress cultures are the proven methods for altering the physiological and biochemical behaviors of cyanobacteria (Allen et al. 2005; Sundaram and Soumya 2011). Therefore, such cultures can elicit metabolism modifications in *A. platensis.* In the normal SOT medium, *A. platensis* produced low cytoplasmic ASNase (0.127 ± 0.107 U). Hence, we modified the SOT medium and culture condition to provide stress to optimize cytoplasmic ASNase production.

In cyanobacteria, nitrogen and iron have a vital role with respect to its photosynthesis (Bauer et al. 1993) and their stresses induce the synthesis of several proteins (Fernandes et al. 1993; Kolodny et al. 2006). Effect of salt stress on growth and photosynthesis of *A. platensis* was reported (Vonshak et al. 1988). Since light is essential to photosynthesis in *A. platensis*, treatment of *A. platensis* under the dark condition in a definite period of time is expected to produce an effect on carbon and nitrogen metabolism in *A. platensis*. Temperature is also one of the stress factors affecting the physiological and biochemical changes in *A. platensis*. It has been reported that the expression level and mRNA stabilization of *desD* gene in *A. platensis* were enhanced by a temperature shift from 35 to 22 °C (Deshnium et al. 2000). On the ground of these previous studies, we chose four factors, N, Fe, NaCl, and dark low temperature as stress factors in this study.

We found that a lack of N and Fe at early stages of growth reduced ASNase production. This result suggested a strong association between N and Fe and ASNase production. An abundance of N in cyanobacteria cells is reserved in proteins found in cyanophycin granules (multi-l-arginyl-poly-l-aspartic acid). A lack of N will decrease the amount of cyanophycin because N is the vital compound in cyanophycin synthesis (Picossi et al. 2004). This phenomenon was confirmed by Allen et al. 1980, who revealed that N deficiency decreases cyanophycin in the cytoplasm. The low yield of ASNase was probably because of low cyanophycin content in the cell. However, a large amount of N (100 % of NaNO_3_) resulted in low ASNase production in SOT normal medium. Hence, N must be available in the medium in sufficient quantities for its usage in cyanophycin and ASNase production.

The other consequence of insufficient amount of N and Fe includes interference in photosynthesis because of abnormal pigments. *A. platensis* cells at all treatments exhibited less green color compared with *A. platensis* cells grown in normal SOT medium (unpublished data). A sign of the development of atypical pigments was indicated by color changes in the cell cultures from green to yellowish. Bleaching of color pigments in cell cultures is a sign of nutrient depletion, particularly N content, which is because of a change in pigment color called “chlorosis” (Collier and Grossman 1992; Sauer et al. 2001). This is followed by a lack of energy supply in the cell because of interferences in photosynthesis. The lacks of iron hampered a complete function of photosynthetic machinery in cyanobacteria including reduced levels of chlorophyll, phycocyanin, phycobilisomes, ferredoxin, flavodoxin and other essential photosynthetic electron transport (Sandmann and Malkin 1983; Sandmann et al. 1990; Ferreira and Straus 1994). Fe stress appeared to induce an energy insufficient condition in the cell throughout the interference in photosynthesis. Exposure of cyanobacteria to high salinity correlated with its time of transition from lag to log phase, demanding higher energy for growth (Vonshak et al. 1988). Cyanobacteria degrade cyanophycin when they require energy. Bacteria exploit cyanophycin for growth and energy because it is abundant reserved source of N, carbon, and energy (Obst et al. 2005). Furthermore, dark environment and lack of energy would trigger *A. platensis* to provide sufficient energy to maintain its metabolism.

Degradation of cyanophycin, which provides an abundance of polypeptides during lack of energy, appeared to cause an effective enhancement of ASNase production. Cyanophycin was degraded in the dark condition where no energy source was supplied. Cyanophycin would be degraded to β-asp-Arg, the main substrate for plant-type ASNase (Richer et al. 1999). It can be inferred that plentiful amounts of β-asp-Arg in the cytoplasm would boost the ASNase production and that β-asp-Arg could be used as an “ASNase inducer”.

In this study, using a Taguchi experimental design, we enhanced ASNase production of the ASNase by approximately 2.17-fold (0.275 ± 0.005 U) compared with production using standard SOT medium with a normal culture (0.127 ± 0.107 U). Further research regarding exploration of more efficient production, purification, characterization and application of the enzyme is needed.

In conclusion, high specific activity of ASNase was found in the cytoplasmic region. Depletion of N and Fe negatively affected ASNase production. NaCl and temperature shock in the dark condition may have contributed to ASNase production because of energy generation through cyanophycin degradation. The link between several experimental factors (Table 3) led us to a new hypothesis that ASNase production is probably induced by multiple factors. We here revealed that combined stresses could be applied to optimize ASNase production in *A. platensis.*

## Electronic supplementary material

Below is the link to the electronic supplementary material.

Supplementary material 1 (DOC 233 kb)
