# Supplementary material for: Combination of environmental stress and localization of l-asparaginase in Arthrospira platensis for production improvement
Source: 3 Biotech. 2014 Apr 13;4(6):647–53. doi: 10.1007/s13205-014-0215-z (PMC4235887; doi:10.1007/s13205-014-0215-z)
Supplement: Supplementary file 1 — Supplementary material 1 (DOC 233 kb) [file 13205_2014_215_MOESM1_ESM.doc]

**Supplementary material 1.**


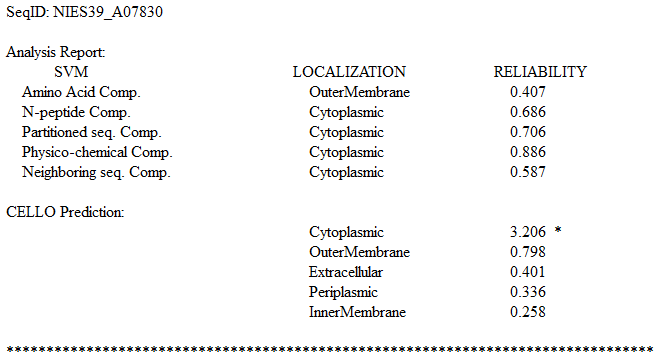


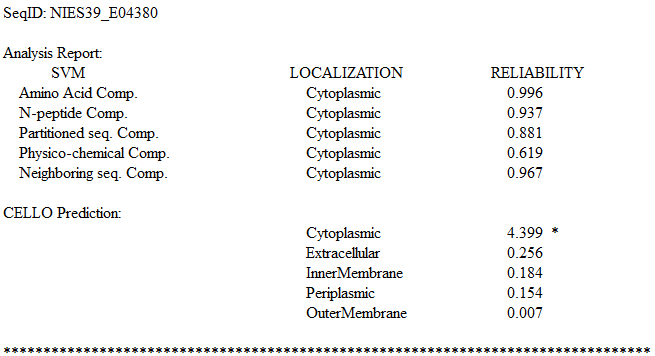


Localization of ASNase, a product of [NIES39_E04380](http://www.genome.jp/dbget-bin/www_bget?arp:NIES39_A07830) and NIES39_E04780 gene based on CELLO ( subcellular localization prediction analysis).


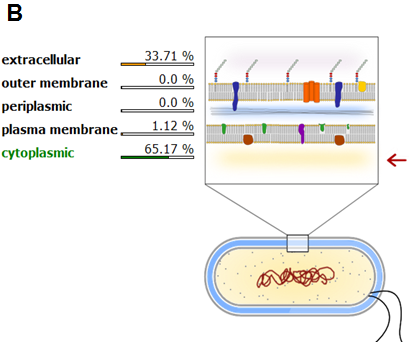

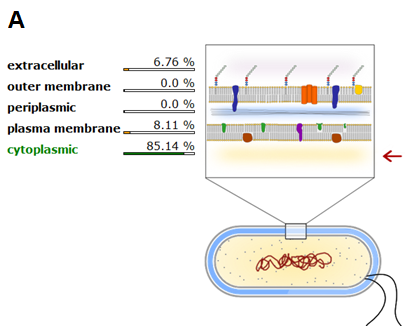


# Localization of ASNase, a product of [**NIES39_E04380**](http://www.genome.jp/dbget-bin/www_bget?arp:NIES39_A07830) (A) and NIES39_E04780 (B) genes based on MetaLocGramN (A meta-predictor of protein subcellular localization for Gram-negative bacteria)


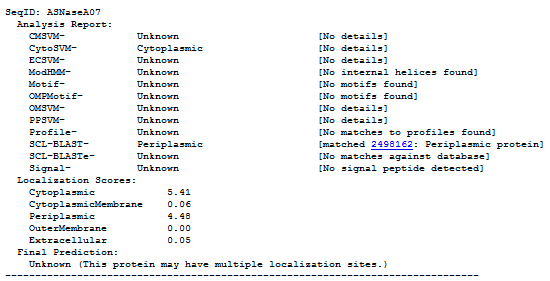


**
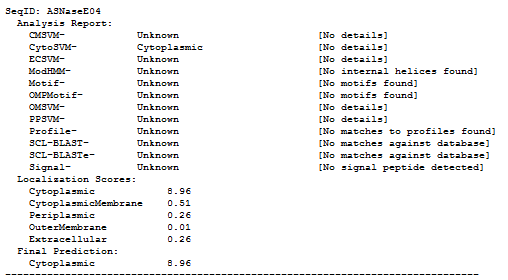
**

Localization ASNase, a product of NIES39_E04780 gene based on PSORTb analysis (Higher score, higher possibility of its existence)
